# P-1423. Barriers to Care: Improving Outpatient Follow-Up for Patients on Long-Term Oral Antimicrobial Therapies

**DOI:** 10.1093/ofid/ofae631.1598

**Published:** 2025-01-29

**Authors:** Heather Cummins, Russell J Benefield, Laura Certain

**Affiliations:** University of Utah and Huntsman Mental Health Institute, Salt Lake City, Utah; University of Utah Health, Salt Lake City, Utah; University of Utah, Salt Lake City, Utah

## Abstract

**Background:**

Best practice for patients on COpAT (Complex Outpatient Antimicrobial Therapy) includes close follow-up once discharged from the hospital. However, some patients discharged with COpAT do not receive monitoring for complications, increasing their risk for re-admission. There is limited analysis regarding patient-specific social determinants of health (SDOH) that may contribute to a reduced follow-up rate. We aimed to determine the percentage of patients discharged on long-term oral antibiotics who received recommended outpatient Infectious Disease (ID) support and potential barriers to follow-up for quality improvement.

**Table 1:**
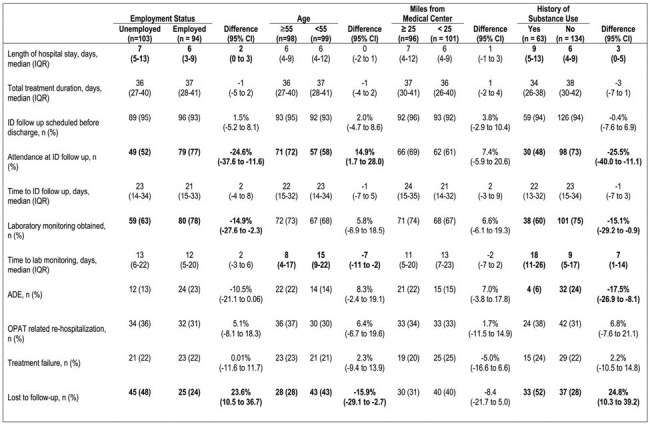
Outcomes associated with social determinants of health Selected social determinants of health associated with specified monitoring and outcomes. Bolded values are considered statistically significant.

**Methods:**

In this single-center cohort, patients discharged between 1/1/2021 and 12/31/2022, on ≥ 2 weeks of oral antibiotics, age > 18 years, with inpatient ID consult, and with recommendations for follow-up in the University of Utah ID Clinic were examined. SDOH included distance from the medical center, age, race/ethnicity, employment status, substance use, homelessness, and history of bipolar disorder/psychoses. We assessed patient outcomes of treatment failure or lost to follow-up within 90 days post-discharge. We also evaluated the time to appointment, lab monitoring, and number of adverse drug events (ADEs) after discharge.

**Results:**

One hundred ninety-seven patients met the inclusion criteria for the cohort. Median times to suggested lab monitoring and ID appointment were 13 and 21 days post-discharge respectively. 71% completed lab monitoring within 90 days of discharge. After 90 days, 19% of patients were lost to follow up and 20% had experienced treatment failure. Those younger than 55, unemployed, or experiencing substance use were more often lost to follow-up. Individuals with substance use or age < 55 had a longer time to lab monitoring. Patients with a history of substance use had fewer ADEs (Table 1).

**Conclusion:**

SDOH that contribute to patient outcomes include a history of substance use and unemployment, suggesting an area of clinical quality improvement. Additionally, those younger than 55 were more often lost to follow-up. Further examination of follow-up protocol for these patients is important to improve patient outcomes.

**Disclosures:**

**Russell J. Benefield, PharmD, BCPS-AQ ID**, Paratek Pharmaceuticals: Grant/Research Support **Laura Certain, MD, PhD**, Zimmer Biomet: Advisor/Consultant

